# Nowcasting Avalanches as Earthquakes and the Predictability of Strong Avalanches in the Olami-Feder-Christensen Model

**DOI:** 10.3390/e22111228

**Published:** 2020-10-28

**Authors:** Jennifer Perez-Oregon, Fernando Angulo-Brown, Nicholas Vassiliou Sarlis

**Affiliations:** 1 Solid Earth Physics Institute, Physics Department, National and Kapodistrian University of Athens, Panepistimiopolis, Zografos, 157 84 Athens, Greece; jnnfr.po@gmail.com; 2 Departamento de Física, Escuela Superior de Física y Matemáticas, Instituto Politécnico Nacional, UP Zacatenco C.P., Mexico City 07738, Mexico; angulo@esfm.ipn.mx; 3 Section of Condensed Matter Physics, Physics Department, National and Kapodistrian University of Athens, Panepistimiopolis, Zografos, 157 84 Athens, Greece

**Keywords:** earthquakes, avalanches, natural time analysis, Olami-Feder-Christensen model, nowcasting earthquakes

## Abstract

Nowcasting earthquakes, suggested recently as a method to estimate the state of a fault and hence the seismic risk, is based on the concept of natural time. Here, we generalize nowcasting to a prediction method the merits of which are evaluated by means of the receiver operating characteristics. This new prediction method is applied to a simple (toy) model for the waiting (natural) time of the stronger earthquakes, real seismicity, and the Olami-Feder-Christensen earthquake model with interesting results revealing acceptable to excellent or even outstanding performance.

## 1. Introduction

Earthquakes (EQs) are complex phenomena with non-trivial correlations in time, space and magnitude (*M*) that have been the subject of a variety of studies that use aspects of statistical physics [1,2,3,4,5,6,7,8,9,10,11,12,13,14,15], see also [16,17] and References therein. Various scaling laws [18] are obeyed by EQs, the most well known of which is the Gutenberg-Richter (GR) law [19] according to which the frequency of EQs versus their magnitude is exponentially distributed resulting in a probability to observe an EQ of magnitude *M* greater than $M_{0}$

(1) $$P\left(M>M_{0}\right)\propto 10^{-bM_{0}},$$

that reflects a power-law distribution for the seismic moment $M_{o}$ (and hence the elastic energy *E* emitted) during an EQ, if we recall that [20]

(2) $$E\propto M_{o}\propto 10^{1.5M}.$$

Within this frame, the observed EQ scaling laws [18] are widely accepted [21,22,23,24] to indicate the existence of phenomena closely associated with the proximity of the system to a critical point.

For the analysis of time series coming from complex systems, a procedure termed natural time analysis [24] was proposed [21,25,26,27] in the beginning of 2000s which enables us to determine when the system approaches the critical point. Natural time analysis also uncovers unique dynamic features hidden behind the time series of complex systems and has found applications in diverse fields, including the Olami-Feder-Christensen (OFC) EQ model [28] (see also Section 2.2), such as the study of EQs [29,30] and the identification of the sudden cardiac death risk [31], compiled in Reference [24], see also References [32,33,34,35] for more recent applications. Natural time, which is unique in its characteristics [36], is currently considered to be the basis for a new methodology to estimate the seismic risk by Turcotte and coworkers [36,37,38,39,40,41,42,43] termed “nowcasting earthquakes”.

EQ nowcasting [37] focuses on describing the current state of fault systems and hence it differs from EQ forecasting [8,44,45,46] in which the probabilities for a future EQ are computed. As suggested by Rundle et al. [37], nowcasting can be used as a basis for forecasting if we have a method to project this current state into the future. In this sense, nowcasting is a prerequisite for forecasting.

Along these lines, the scope of the present paper is to suggest a procedure to generalize nowcasting into forecasting by introducing a prediction scheme based on the knowledge of the current state of the system as it is reflected by the waiting (natural) time between strong events. In the next section, we briefly present the two basic ingredients of this scheme which are natural time (Section 2.1) and nowcasting (Section 2.3) as well as introduce, in Section 2.4, a toy model for which we formulate the proposed prediction scheme (see Section 2.4.2). The results follow in Section 3 where we apply it to a recent example of a magnitude M${}_{w}$6.8 EQ in Greece—for which nowcasting has already appeared [47] in the literature—and to the case of the OFC EQ model [28]. Section 4 provides a discussion of the results found while the conclusions are presented in Section 5.

## 2. Methods

### 2.1. Natural Time Analysis Background

**Figure 1:**
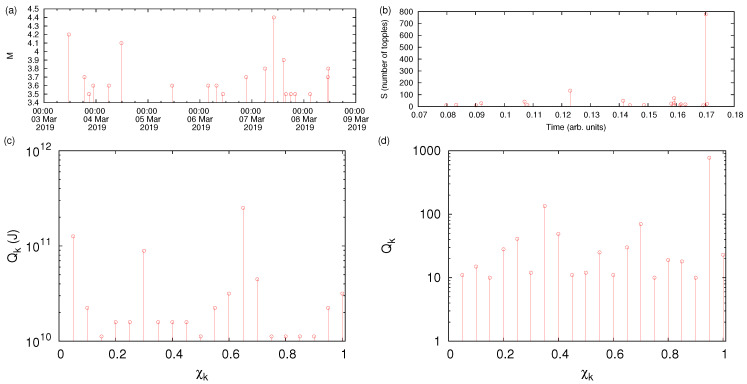
A series of (**a**) EQs or (**b**) avalanches when read in natural time are represented by the panels (**c**,**d**), respectively.

In a time series comprised of *N* individual events, for example EQs with magnitude greater than or equal to a threshold $M_{\lambda }$ or avalanches with size $S(\geq S_{c})$, the natural time [21,24,25,48] associated with the *k*-th event is given by $\chi_{k}=k/N$. At this point, we have to clarify that the term avalanches is a general term to refer to synthetic events made for instance by the OFC model (see below) or other systems (e.g., sandpiles, solar flares) that relax through spatially distributed changes that involve a large number of agents and in which the build-up to instability is slow, while relaxation is fast accompanied by avalanche-like, bursty energy release that may cover a broad range of scales, e.g., see [49]. Usually, the pair $\left(\chi_{k},Q_{k}\right)$ is studied [21,24,25,48], where $Q_{k}$ is a quantity proportional to the energy emitted during the *k*-th event. For example in the case of EQs, it may be considered—by virtue of Equation (2)—proportional to the seismic moment [21,48,50] $M_{o}$ (cf. the energy available for generation of seismic waves is [20]$\left(\Delta \sigma /2\mu_{o}\right)M_{o}$, where Δσ is the stress drop and $\mu_{o}$ the shear (rigidity) modulus of the fault) while for avalanches $Q_{k}$ is considered [24,51] proportional to the size *S*. Figure 1 shows how a series of EQs or avalanches are read in natural time.

The pair $\left(\chi_{k},Q_{k}\right)$ is studied by considering the normalized energy for the *k*-th event $p_{k}=Q_{k}/\sum_{n=1}^{N}Q_{n},$ where $p_{k}$ can be also considered as a probability distribution [33,52]. The main concept behind the introduction of natural time was that the state of the complex system changes upon the occurrence of each event and that the distribution $p_{k}$ contains important information for the process [24].

It was found [24,25,53,54,55,56] that the variance of natural time

(3) $$\kappa_{1}\equiv \sum_{k=1}^{N}\chi_{k}^{2}p_{k}-{\left(\sum_{k=1}^{N}\chi_{k}p_{k}\right)}^{2},$$

with respect to the distribution $p_{k}$ is a very useful quantity that may identify the approach of the complex system studied to a critical point. For example for critical systems (such as the 2D or 3D Ising model, XY model, 3-state Potts model, etc.) and when from a high temperature state they rapidly approach the critical temperature and hence criticality, the validity of the following equation has been shown [54]

(4) $$\kappa_{1}\approx 0.07;$$

where $\kappa_{1}$ can be also considered to be the order parameter of a phase transition which abruptly changes just before the mainshock (new phase) [53].

Another useful quantity in natural time analysis is the entropy *S* given by [21,26,57]

(5) S=〈χlnχ〉−〈χ〉ln〈χ〉,

where the brackets $\langle \ldots \rangle \left(\equiv \sum_{k=1}^{N}\ldots p_{k}\right)$ denote averages with respect to the distribution $p_{k}$. The entropy *S* is a dynamic entropy that exhibits [58] positivity, concavity and Lesche [59,60] experimental stability. Any valid definition of entropy must be capable of distinguish between reversible and irreversible processes. The natural time entropy has this property; that is, for irreversible processes, it is not invariant under time reversal and this permits important applications in several fields [47,51]. Another very important version of entropy is the so-called multi-scale entropy that is able to distinguish subtle levels of complexity and different degrees of irreversibility also using both entropy in the direction of time and entropy in the reverse direction [61,62].

### 2.2. The Olami-Feder-Christensen Earthquake Model

The spring-block model was developed by Burridge and Knopoff [63] in 1967 with the purpose of explaining the empirical law given by Gutenberg and Richter on the distribution of EQs according to their magnitude [19]. Later, this model was mapped into a two dimensional non-conservative cellular automaton by Olami, Feder, and Christensen [28] which simulates the relative and frictional movement between two tectonic plates which is the main process which causes EQs.

In this way, the spring-block model is a two-dimensional dynamic system with open boundary conditions (OBC), in which the sites at the boundary distribute energy to the outer sites (that do not belong explicitly to the square lattice) and since these sites cannot topple, their energy is removed or lost at the boundary. The model considers two plates in which the bottom plate is made up of a series of blocks joined to each other by elastic springs. Those blocks are also connected by springs to the upper plate that moves with a small but constant speed *V*, allowing interaction between both plates, so that the movement of the top plate transmits energy to the block system of the bottom plate. When the accumulated energy of one of the blocks reaches or exceeds a given threshold (the maximal static friction), this block will slip and then it is said that the site *topples*, beginning to transmit its energy (cf. theoretical [64] and experimental [65] studies have provided valuable information on significant precursor dynamics before the occurrence of the transition to sliding) to its four neighbors (left, right, front, and rear). Thus, this will begin a possible chain reaction that will represent a synthetic EQ (if only one site topples, the size of the EQ will be 1). Indeed, the number of toppled sites represents the size of the synthetic seism. In this model it is assumed that the static friction threshold has the same value in all blocks. If energy input occurs in discrete steps instead of continuous and if thresholds are random but not quenched, quasiperiodicity emerges combined with power-laws [66].

Despite some limitations of the OFC model, where the criticality of the model is perhaps the most worrisome (it seems that the self-organized criticality behavior of the model is lost by introducing variations in the model rules [67,68] for example by replacing the OBC with periodic boundary conditions [69], by introducing frozen noise in the local degree of dissipation [70], i.e., by considering at each site a different $\alpha_{i}=\alpha +\delta_{i}$ instead of just α, where $\delta_{i}$ is a random number coming from the uniform distribution in the interval $\left[-\delta ,\delta \right]$ which is kept constant throughout the simulation, or in its threshold value [71], and by including lattice defects [72]), the OFC model has been able to successfully reproduce qualitative important features observed in real seismicity such as the GR law [19], the stair graphs for the cumulative frequency [49,73,74,75,76,77,78] and also very recently, the Ruff–Kanamori diagram [79,80]. Also as far as EQ predictability [81] or Omori law [82,83] are concerned, the OFC model appears to be closer to reality than others [84].

In the present work, we follow without any addition or modification the non-conservative cellular automaton proposed by OFC [28]. The OFC model runs considering a square L×L lattice where on each block acts a force $F_{i,j}$, with *i* and *j* integers between 1 and *L*. The steps that the cellular automaton must follow [28] are the following:

(1) All the sites or blocks of the square lattice should be initialized making an assignment of each one of them with a continuous random variable between zero and a threshold force $F_{th}$, representing the local energy of the block.

(2) If a block topples the force $F_{i,j}$ relaxes according to the rules

(6) $$\begin{array}{ccc} F_{i\pm 1,j} & \to & F_{i\pm 1,j}+\alpha F_{i\pm 1,j}, \end{array}$$

(7) $$\begin{array}{ccc} F_{i,j\pm 1} & \to & F_{i,j\pm 1}+\alpha F_{i,j\pm 1}, \end{array}$$

(8) $$\begin{array}{ccc} F_{i,j} & \to & 0 \end{array}$$

where α is related to the ratio of the elastic constants of the springs, i.e., $\alpha =\frac{K_{h}}{4K_{h}+K_{v}}$, where $K_{h}$ and $K_{v}$ are the elastic constants for the springs either between the blocks themselves or between a block and the upper plate, respectively.

(3) Step 2 should be repeated until the synthetic event has completely evolved, i.e., the condition $F_{i,j}<F_{th}$ is satisfied for every $F_{i,j}$.

(4) A global perturbation should be performed, this means add $F_{th}-F_{max}$ to all sites, where $F_{max}$ is the largest force in the current state of the system. Then go to step 2 until the event has completely evolved.

As it has been said before, the number of toppled sites (or topplings) defines the size *S* of a synthetic EQ. This quantity *S* is the one used as $Q_{k}$ in the natural time analysis [24,51,85]. The coupling parameter or elastic ratio α can take values from zero to 0.25 and represents the ratio of energy that the toppled site transmits to its neighbors. In this way, α=0.25 corresponds to the conservative case meaning that the neighbors of the toppled site will receive all the energy of the toppled site, while a smaller value of α means some degree of dissipation (the smaller the α, the bigger the dissipation). Two underlines about the model should be pointed out: once the size of the square lattice *L* is fixed, the only remaining parameter of the model is the elastic ratio α and excluding the initial condition the model is deterministic. As said, the aforementioned boundary conditions were OBC, but of course, the model accepts other types of boundary conditions such as free boundary conditions (FBC) in which α varies locally following the expression: $\alpha_{ij}=1/\left(n_{ij}+K\right)$, where $n_{ij}$ is the number of nearest neighbors of the ij block; that is, $n_{ij}=4$, for the blocks in the bulk of the lattice, $n_{ij}=3$, for the blocks on the edges and $n_{ij}=2$, for the four blocks at the corners of the lattice, the elastic constant of the superior leaf springs measured relative to the other springs between blocks is symbolized by *K* [83], i.e., $K=K_{v}/K_{h}$ (clearly, the OFC model is not conservative for K>0 for which $\alpha_{ij}<0.25$ in the bulk). In both OBC and FBC cases, the blocks on the lattice border receive energy only from three or two neighbors, which causes that, on average, these blocks topple less frequently than the sites in the interior, this behavior leads to patches of sites with similar energy. In this way, patching proceeds from the boundaries inward [86,87]. Lastly, periodic boundary conditions (PBC) could also be imposed, but since they destroy the criticality of the system [69], it is not very useful to employ this type of boundary conditions. The existence of avalanches of all sizes is due to the dynamics of the model.

A thorough numerical and analytical study in Reference [84] showed finally that the observed “power-laws” are *dirty* power-laws, which appear as power-laws over a wide range of parameters and over a few decades of avalanche sizes, while the “true” analytical form is no power-law.

### 2.3. Earthquake Nowcasting

As mentioned in the Introduction, nowcasting EQs is a method for the determination of the current state of a fault system in the sense that it determines [37] the progress in the EQ cycle. To achieve this determination, one employs an EQ catalog to calculate from the ‘small’ EQs, which are defined as those with magnitude $M<M_{\sigma }$ but above a threshold $M_{\lambda }$, i.e., of magnitude $M\in [M_{\lambda },M_{\sigma })$, the level of hazard for ‘strong’ $M\geq M_{\sigma }$ EQs. The lower threshold $M_{\lambda }$ is typically the completeness threshold of the EQ catalog used [37], while the EQ catalogs used [36,37,38,47] are global seismic catalogs such as the Advanced National Seismic System Composite Catalog or the United States National Earthquake Information Center (NEIC) PDE catalog [88]. For such global catalogs, a magnitude threshold $M_{\lambda }=4.0$ has been considered [36,38] for applications in EQ prone areas outside United States, such as those in Greece, Japan, and India. By employing the natural time concept, one counts the number $n_{i}$ of small EQs that occur after a strong EQ. In other words, $n_{i}$ is the waiting (natural) time or interoccurrence natural time. The current number n(t) of small EQs since the occurrence of the last strong EQ is compared with the cumulative distribution function (CDF) of the interoccurrence natural time $Prob(n_{i}<n)$. For such an estimation of $Prob(n_{i}<n)$, it should be ensured [37] that we have enough data to span at least 20 or more strong EQ cycles, thus the size of the available EQ catalog together with the selection of $M_{\lambda }$ and $M_{\sigma }$ should be made carefully to comply with this rule. In nowcasting EQs [37], the earthquake potential score (EPS) equals the CDF value,

(9) $$\mathrm{EPS}=Prob[n_{i}<n\left(t\right)],$$

and is a measure of the level of current hazard. As mentioned in the Introduction, nowcasting EQs were found useful in many applications [36,37,38,39,40,41,42,43] including the highly important estimation of seismic risk to various cities of the world. In this application, EQs, reported as mentioned in a Global catalog, with depths smaller than a given value *D* within a large area are studied [38] in order to obtain the CDF of $n_{i}$. Then the number $\tilde{n}$ of small EQs around a city, i.e., those occurring within a circular region of epicentral distances r<R with hypocenters shallower than *D*, is counted since the occurrence of the last strong EQ in this circular region. Based on the ergodicity of EQs that was proven [89,90,91] by using the metric published in References [92,93], Rundle et al. [38] suggested that the seismic risk around a city can be estimated by using the EPS corresponding to the current value of $\tilde{n}$ by setting $n\left(t\right)=\tilde{n}$ in Equation (9).

### 2.4. A Simple Log-Normal Model for the Earthquake Potential Score

#### 2.4.1. Definitions

**Figure 2:**
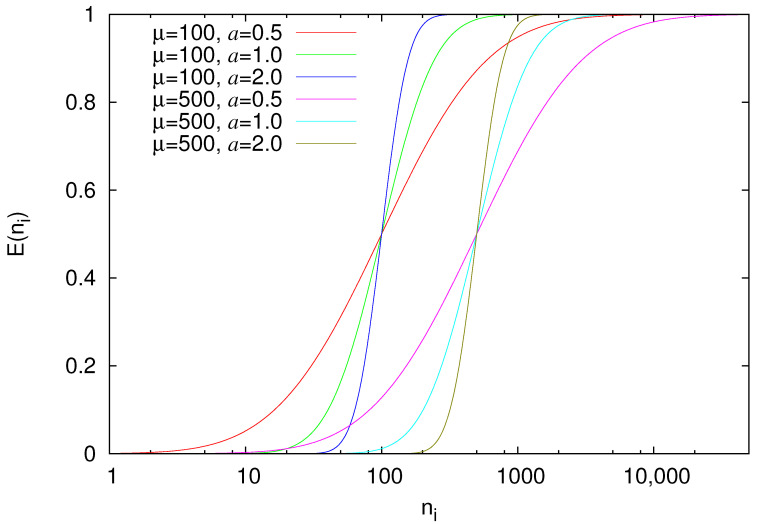
EPS $E(n_{i})$ as function of $n_{i}$ for various values of the parameters μ(=100,500) and a(=0.5,1,2) of Equation (10).

The Log-normal distribution has been used [36] among others for describing the statistics of the interoccurrence natural time $n_{i}$ between strong EQs, i.e., those with magnitude *M* larger than a threshold $M_{\lambda }$, $M\geq M_{\lambda }$, and have been found to show the best fit to the observed natural time data, see Table 3 of [36]. Since such a model is also an easily accessible one, we employ it here. Specifically, we condider a Log-normal model according to which the EPS $E(n_{i})$, that equals the aforementioned CDF of $n_{i}$, is given by

(10) $$E\left(n_{i}\right)=\frac{1+\mathrm{erf}[a\ln\left(n_{i}/\mu \right)]}{2},$$

where erf(x) is the error function of *x* (for its definition see Equation (7.1.1) of Reference [94]). The parameters *a* and μ are related with the standard deviation of the underlying Log-normal distribution ($\sigma =1/\sqrt{2}a$) and the median of $n_{i}$ (cf. E(μ)=50%), respectively. Figure 2 provides a few examples of the EPS as a function of $n_{i}$ for various values of *a* and μ.

Earthquake nowcasting [36,37,38,39,40,41,42,43] uses EPS as the basis to estimate the seismic risk, as already mentioned. Here, we will attempt to use the EPS statistics as a possible EQ prediction method that will be evaluated by means of the Receiver Operating Characteristics (ROC) technique (e.g., see Reference [95]). We assume that if we set the alarm ON then the next EQ is expected to be a strong EQ of magnitude $M\geq M_{\sigma }$. If this is true then we have a hit (true positive prediction), otherwise we have a false alarm (false positive prediction). If the alarm is OFF and the next EQ is a strong one, we have a miss (false negative prediction) while if it is not strong we have a true negative prediction. ROC quantifies the quality of a prediction scheme by means of ploting the True Positive rate (TPr), or hit rate, versus the False Positive rate (FPr), or false alarm rate. TPr is simply the ratio of True Positives (TP) over the total number of Positive (*P*) cases (strong EQs), i.e.,

(11) TPr=TP/P,

while the False Positive rate is the ratio of the False Positives (FP) over the total number of negative cases (*Q*) (i.e., the number of small $M<M_{\sigma }$ EQs),

(12) FPr=FP/Q.

**Figure 3:**
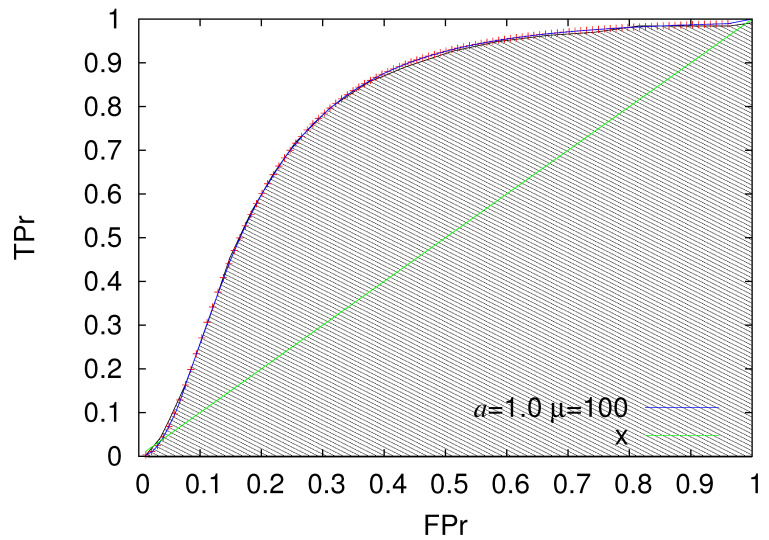
The ROC for the model $E(n_{i})$ of Equation (10) for a=1 and μ=100 for various values of *l* and *L* (red plus symbols). The blue solid line has been drawn by considering the maximum TPr value obtained from any (*l*,*L*) combination every 0.001 of FPr. The AUC that characterizes the predictability of the a=1, μ=100 Log-normal model of Equation (10) is depicted by the line shaded area. The first diagonal (TPr = FPr) is drawn with the green line as guide to the eye.

The quality of a prediction scheme is evaluated [95] by means of the area under the ROC curve (AUC) (i.e., the area under the ROC curve and above the x-axis in Figure 3). For random predictions, AUC has been shown [96] to follow Mann-Whitney [97] statistics while the corresponding ROC fluctuates around the first diagonal TPr = FPr, for more details see, e.g., Reference [98].

#### 2.4.2. A Prediction Scheme Based on Nowcasting

Let us now return to our simple prediction scheme which can be based, for example, on Equation (10). In other words, we assume that the analysis of a global EQ catalog (such as those mentioned in Section 2.3) with appropriate threshold $M_{\lambda }$ targeting to strong EQs with $M\geq M_{\sigma }$ has led to an EPS such as those depicted in Figure 2 and on the basis of this observation we employ the following prediction scheme: We set the alarm ON when $n_{act}$, i.e., the actual number of small ($M\geq M_{\lambda }$) EQs that occured after the last strong EQ, is greater than or equal to *l*, $n_{act}\geq l$, and keep the alarm ON until $n_{act}$ is smaller than or equal to *L*, $n_{act}\leq L$. Obviously, L≥l and by varying both *L* and *l* we can obtain various values of TPr and FPr leading to the ROC shown in Figure 3. In principle, the values of *l* and *L* can be fortuitous as far as L≥l and they both lie within the range of variation of $n_{act}$ as the latter is estimated by means of the EPS, e.g., see Figure 2. As we will see in Section 2.4.3, we propose the selection *l* from μ/10 up to μ and *L* from μ+5 up to a value $L_{max}$ which corresponds to a high percentage of EPS, e.g., $E(L_{max})=99\%$, because the investigation of the ROCs obtained from such combinations of *l* and *L* may reveal for each value of FPr an optimum selection of *l* and *L* that maximizes AUC. The value of $L_{max}$ can be related to the range $\Delta M(=M_{\sigma }-M_{\lambda })$ as will be also discussed in Section 2.4.3 below.

#### 2.4.3. Evaluation of the Prediction Scheme

Since the hit rate is just the percentage increase between *l* and *L* by using Equation (10) we obtain

(13) $$\mathrm{TPr}=E\left(L\right)-E\left(l\right)=\frac{\mathrm{erf}[a\ln(L/\mu )]-\mathrm{erf}[a\ln(l/\mu )]}{2}.$$

We now turn to the estimation of FPr. According to Equation (12), we can decompose it into two ratios:

(14) $$\mathrm{FPr}=\frac{\mathrm{FP}}{Q}=\left(\frac{\mathrm{FP}}{P}\right)\left(\frac{P}{Q}\right).$$

Since EQ data, follow the GR law of Equation (1) one can show (see Equation (2) of Reference [37]) that

(15) $$\frac{P}{Q+P}=10^{b(M_{\lambda }-M_{\sigma })}$$

where $M_{\lambda }$ is the threshold magnitude above which an EQ is included in the catalog used for estimating $n_{i}$. Equation (15) leads to

(16) $$\frac{P}{Q}=\frac{10^{b(M_{\lambda }-M_{\sigma })}}{1-10^{b(M_{\lambda }-M_{\sigma })}}\approx 10^{b(M_{\lambda }-M_{\sigma })}.$$

where in the last part of Equation (16) we assumed $1\gg 10^{b(M_{\lambda }-M_{\sigma })}$ since usually $M_{\lambda }-M_{\sigma }\leq -3$ and b≈1. The other term of Equation (14), i.e., the ratio FP/P, can be also estimated on the basis of $E(n_{i})$ on the following grounds: FP/P is the weighted average of the number of false positive alarms, i.e., it equals (L−l+1) with a probability equal to that of $n_{i}$ to exceed *L* that is [1−E(L)], (L−l) with the probability $n_{i}=L$ that is [E(L)−E(L−1)], (L−l−1) with the probability $n_{i}=L-1$ that is [E(L−1)−E(L−2)], etc. Thus, we have

(17) $$\frac{\mathrm{FP}}{P}=\left(L-l+1\right)\left(1-E\left(L\right)\right)+\left(L-l\right)\left(E\left(L\right)-E\left(L-1\right)\right)+\ldots +1\left(E\left(l+1\right)-E\left(l\right)\right)$$

which by rearranging terms

(18) $$\begin{array}{ccc} \frac{\mathrm{FP}}{P} & = & (L-l+1)-(L-l+1)E(L)+\\ & + & (L-l)E(L)-(L-l)E(L-1)+\\ & + & (L-l-1)E(L-1)-(L-l-1)E(L-2)+\ldots \\ & + & 2E(l+2)-2E(l+1)+\ldots \\ & + & E(l+1)-E(l) \end{array}$$

leads to

(19) $$\frac{\mathrm{FP}}{P}=L-l+1-\sum_{n=l}^{L}E\left(n\right).$$

We observe that Equations (14), (16), and (19) can be combined for the estimation of FPr not only in the Log-normal case of Equation (10) but also in any other theoretical or experimental estimate of the EPS versus $n_{i}$ because they do not depend on the explicit form of $E(n_{i})$.

We now return to our Log-normal prediction model, as said, we set the alarm ON when $n_{i}=l$ and set it OFF when either $n_{i}=L$ or the next EQ is a strong one. In Figure 3, we depict the ROC curves obtained when varying *l* from μ/10 to μ and *L* from l+5 to $L_{max}$ and mark the corresponding operation points on the ROC diagram (see the red plus symbols in Figure 3). The selection of $L_{max}$ effectively determines the maximum TPr in each case according to Equation (13). In Figure 3, we selected—in order to approach the TPr value of unity—such an $L_{max}$ so that $E(L_{max})=99\%$ or equivalently, when using Equation (10),

(20) $$L_{max}=\mu \exp\left(c/a\right)$$

with $c=c_{0.99}=1.65$ (cf. [1+erf(1.65)]/2=0.99). Moreover, the estimation of FPr requires some value of P/Q to be assumed. A reasonable (and self-consistent) value is

(21) $$\frac{P}{Q}=\frac{1}{L_{max}},$$

since such a selection reflects that with probability equal to one after $L_{max}$ EQs a strong EQ completes an EQ cycle, as discussed in the second paragraph of the Introduction of Reference [37]; see also Reference [43].

To estimate the quality of the predictions of such a model, every 0.001 of FPr we estimate the maximum value of TPr, determine the corresponding values *l*(FPr), *L*(FPr), and construct the blue solid line of Figure 3. The AUC of this blue solid line of Figure 3 provides a measure of the predictability of EQs based on the present Log-normal model. It is obvious that when using the values l=l(FPr) and L=L(FPr), an application of this prediction scheme to any data satisfying Equation (10) will lead to the AUC just calculated.

**Figure 4:**
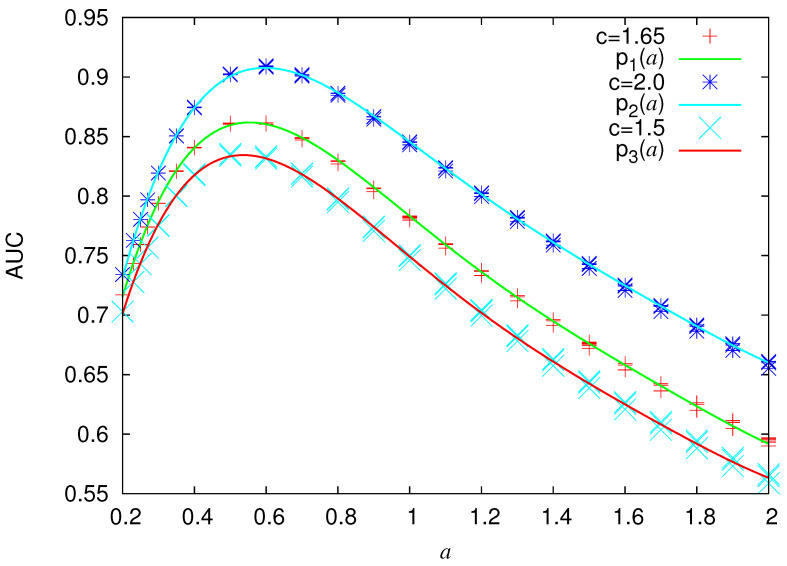
AUC as a function of *a* for three selections of the parameter *c* of Equation (20), i.e., c= 2, 1.65, and 1.5 (from top to bottom). The scattered points for each *a* value correspond various values of μ in the range 30 to 300. The expression of the least squares 5-th order polynomials are $p_{1}\left(a\right)=0.4045+2.1937a-3.696a^{2}+2.699a^{3}-0.948a^{4}+0.13a^{5}$, $p_{2}\left(a\right)=0.3989+2.307a-3.719a^{2}+2.651a^{3}-0.917a^{4}+0.1246a^{5}$, $p_{3}\left(a\right)=0.4010+2.134a-3.674a^{2}+2.721a^{3}-0.967a^{4}+0.1342a^{5}$.

**Figure 5:**
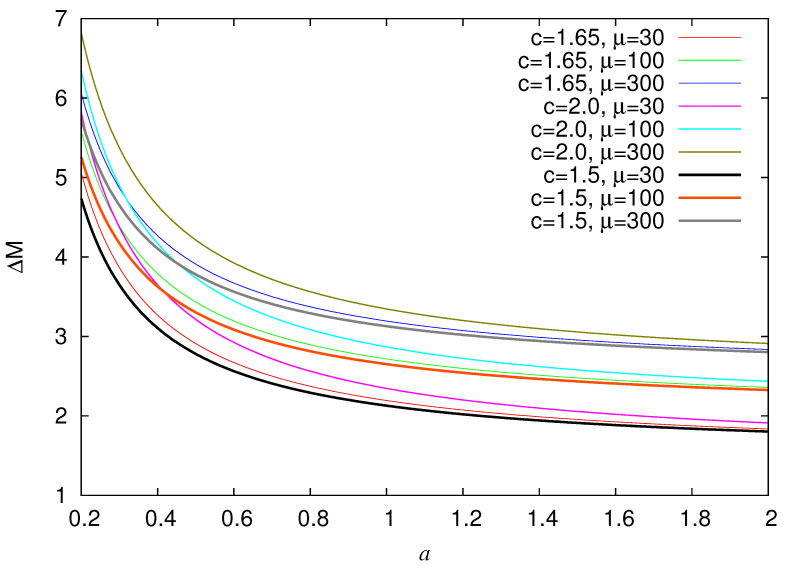
The value of ΔM for various values of *a* and μ when assuming b=1 in Equation (16).

Since the model is parametrized by *a* and μ, we depict in Figure 4 the estimated AUC for various values of these parameters. An inspection of this figure reveals that AUC slightly depends on μ while it mainly depends on *a*. This is not unexpected because *a* quantifies in the Log-normal distribution the width of the probability density peak around μ. The results show that in the range of *a* from 0.2 to around 1.2 AUC values of more than 0.70 can be achieved with maximum value close to 0.90 (cf. AUC values between 0.7 and 0.8 are considered [99] acceptable, between 0.8 and 0.9 excellent, while values higher than 0.9 correspond [99] to outstanding). To connect these theoretical results to real seismicity by using Equations (16), (20), and (21), we depict in Figure 5 the estimated $\Delta M(=M_{\sigma }-M_{\lambda })$ between the smallest magnitude $M_{\lambda }$ considered in the EQ catalog and the smallest magnitude $M_{\sigma }$ of the strong EQs in the case b=1. We observe that reasonable values of ΔM are obtained for *a* values in the wide range 0.2 to 1.2.

## 3. Results

### 3.1. Applications of Log-Normal Model to Real Seismicity

**Figure 6:**
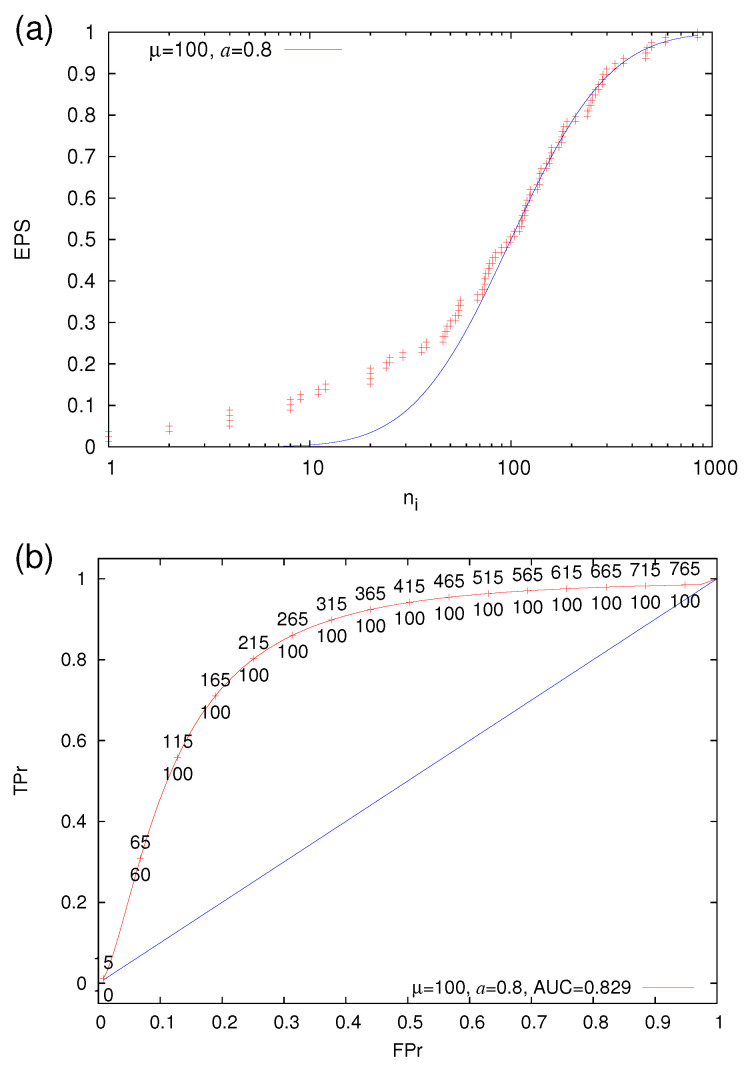
(**a**) The EPS obtained when EQ nowcasting [47] (with $M_{\lambda }=4.0$ and $M_{\sigma }=6.0$) before the 25 October 2018 M${}_{w}$6.8 EQ fitted with a Log-normal model with a=0.8 and μ=100. (**b**) The ROC (red curve) obtained for this Log-normal case (a=0.8, μ=100) is shown together with corresponding optimum selection of *l*(FPr) and *L*(FPr) (numbers below and above the red curve, respectively); AUC is 0.829 which is considered [99] excellent. The first diagonal (TPr = FPr) is drawn with the blue line as guide to the eye.

If someone identifies that an experimental EPS is well fit by Equation (10) then he/she can select an FPr value at which the prediction scheme should operate according the trade-off between the damage cost and false alarm cost (as described in Reference [95]). Then, by varying *l* and *L* and selecting c=1.65 as described in the Section 2.4.3, he/she can find the maximum TPr (see the caption of Figure 3) which is obtained, as mentioned, for the specific values l=l(FPr) and L=L(FPr), see, e.g., the numbers shown in Figure 6b. Such a real world example is the one discussed in Reference [47] concerning a strong EQ of moment magnitude M${}_{w}$6.8 that occurred [100] on 25 October 2018 22:55 UTC at an epicentral distance around 133 km SW of the city of Patras, Western Greece. The application of earthquake nowcasting has shown [47] that $n_{i}=212$ (see Figure 4 of Reference [47]) and the EPS (determined by using the NEIC PDE catalog from 1975 within the large area $N_{29}^{47}E_{12}^{35}$, see Section 3.4 of [47], included 78 strong EQ cycles that secure reliable statistics as mentioned in Section 2.3) is shown in Figure 6a. In this figure, we also show that such an experimental EPS curve can be approximated by a Log-normal model with a=0.8 and μ=100. In Figure 6b, the optimum ROC obtained by the procedure described in the previous Section 2.4.3 shows that if someone operated with l=100 and L=215, he/she would have predicted this strong EQ (since $n_{i}=212\in \left[100,215\right]$) with an FPr close to 0.25 (cf. the corresponding TPr is 80%).

### 3.2. Predictability of the Olami-Feder-Christensen Model Based on the Time-Series of Avalanches

**Figure 7:**
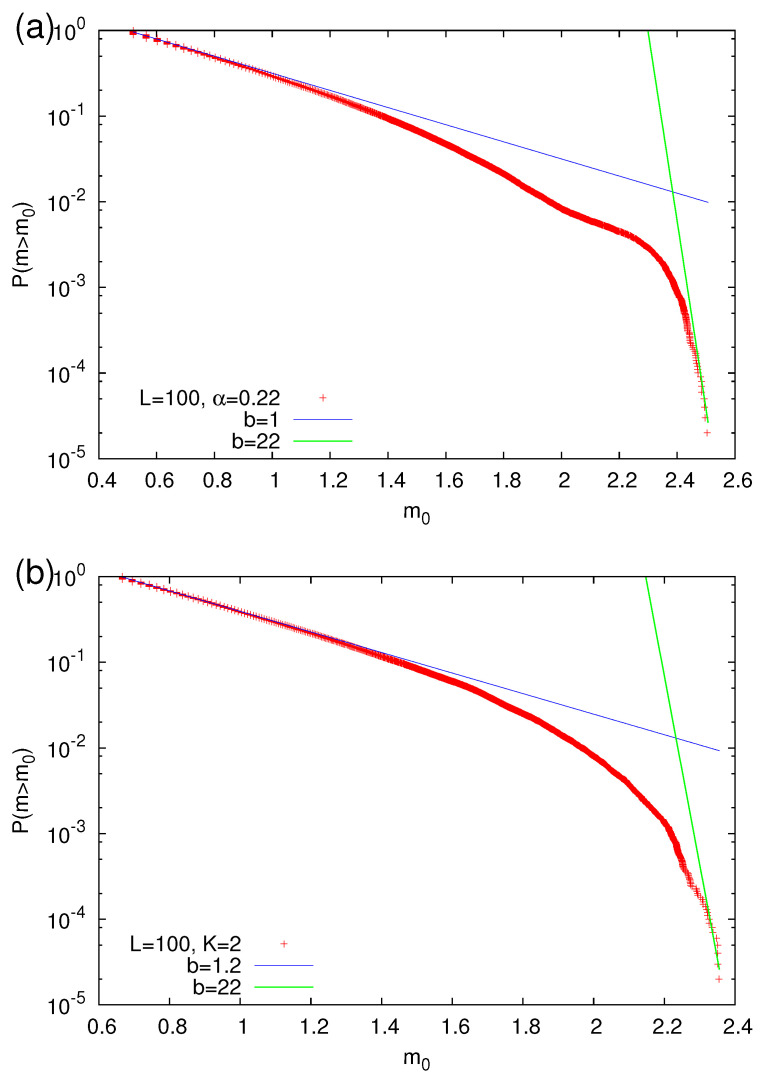
Approximate GR law distributions (reflecting the aforementioned dirty power-law) for the OFC EQ model with values of the linear dimension L=100 of the square lattice and parameters (**a**) α=0.22 or (**b**) K=2. The green lines which are extremely steep have been drawn as a guide to the eye in order to visualize the deviation from the GR law at large magnitudes.

**Figure 8:**
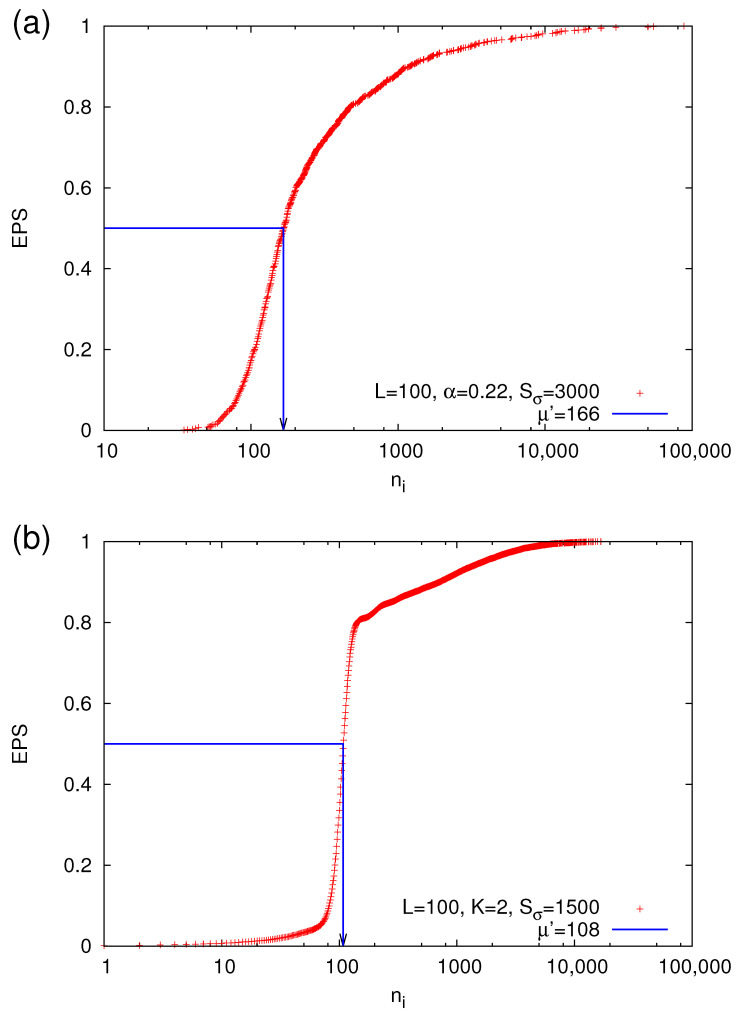
EPS versus $n_{i}$ for the OFC EQ model with linear dimension L=100 of the square lattice and parameters (**a**) α=0.22 or (**b**) K=2. The strong avalanche magnitude threshold is $m_{\sigma }=2.318$ and 2.117, respectively (equivalently the corresponding threshold sizes are $S_{\sigma }=$ 3000 and 1500). The blue lines show the determination of the median $\mu^{\prime }$, i.e., EPS$(n_{i}\geq \mu^{\prime })=$ EPS$(n_{i}\leq \mu^{\prime })=50\%$, in each case.

The aforementioned *dirty* power-laws of the OFC EQ model (see Section 2.2) give rise to GR law distributions which exhibit a lack of large size avalanches such as those shown in Figure 7. We now focus on the application of the prediction scheme of Section 2.4.2. Based on the fact that for EQs the emitted energy *E* is related to the (moment) magnitude *M* by Equation (2), we select for the study of the OFC EQ model the following magnitude (*m*) scale:

(22) $$m=\frac{2}{3}\log_{10}\left(S\right),$$

where *S* is the size (number of topples) of the avalanche. In view of this definition, the GR law of Equation (1) can be seen in Figure 7 for two cases: one corresponding to OBC and the other to FBC. Using a lower magnitude threshold $m_{\lambda }$, we define a natural time step when an avalanche of magnitude exceeding or equal to $m_{\lambda }$ occurs. A strong avalanche is defined as a (rare) event of $m\geq m_{\sigma }$. Selecting a value for $m_{\sigma }$, we obtain the EPS functions shown in Figure 8 (a size threshold of 6 or 10—with corresponding $m_{\lambda }=0.519$ or 0.667—has been used for OBC or FBC, respectively).

**Figure 9:**
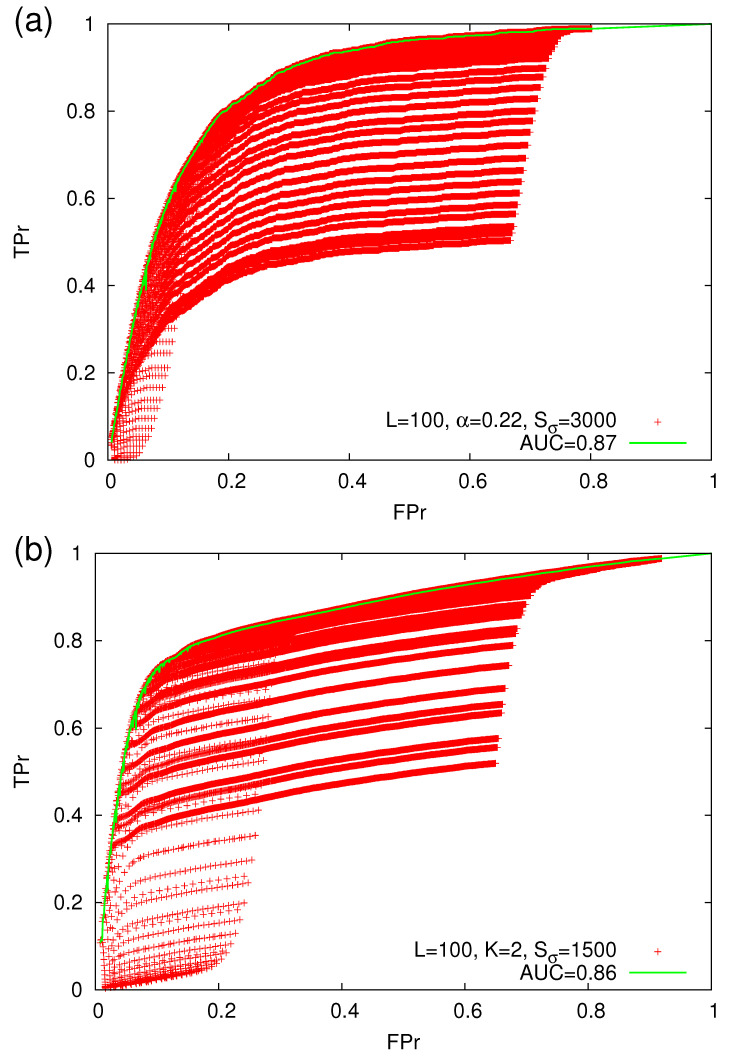
ROC curves (green lines) obtained for the OFC EQ model with values of the linear dimension L=100 of the square lattice and the parameters (**a**) α=0.22 or (**b**) K=2. The strong avalanche magnitude threshold is $m_{\sigma }=2.318$ and 2.117, respectively (equivalently the corresponding threshold sizes are $S_{\sigma }=$ 3000 and 1500). The red plus symbols correspond to the operating points obtained for various values of *l* and *L*.

By employing the prediction method discussed in Section 2.4.2, we turn the alarm ON when $n_{i}=l$ and switch it OFF when either a strong avalanche of $m\geq m_{\sigma }$ occurs in the next natural time step or $n_{i}=L$. Then, we determine by direct evaluation the values of FPr and TPr and depict them with the red plus symbols in Figure 9. This figure has been constructed by varying *l* from $\mu^{\prime }/10$ to $\mu^{\prime }$ (where $\mu^{\prime }$ is now the median of the EPS, i.e., EPS$(n_{i}\geq \mu^{\prime })=$ EPS$(n_{i}\leq \mu^{\prime })=50\%$, which can be determined by means of Figure 8) and *L* from l+10 up to the value of $n_{i}$ corresponding to the 99% EPS percentile. Such a procedure leads to the cloud of the operating points shown by the red plus symbols in Figure 9. Using these points, we can optimize the results (see the green lines in Figure 9), in a fashion similar to that used in Section 2.4.3, i.e., by selecting *l*(FPr) and *L*(FPr) that give rise to the ROC that maximizes TPr every 0.001 step of FPr leading to an optimal (maximum) value of AUC.

**Figure 10:**
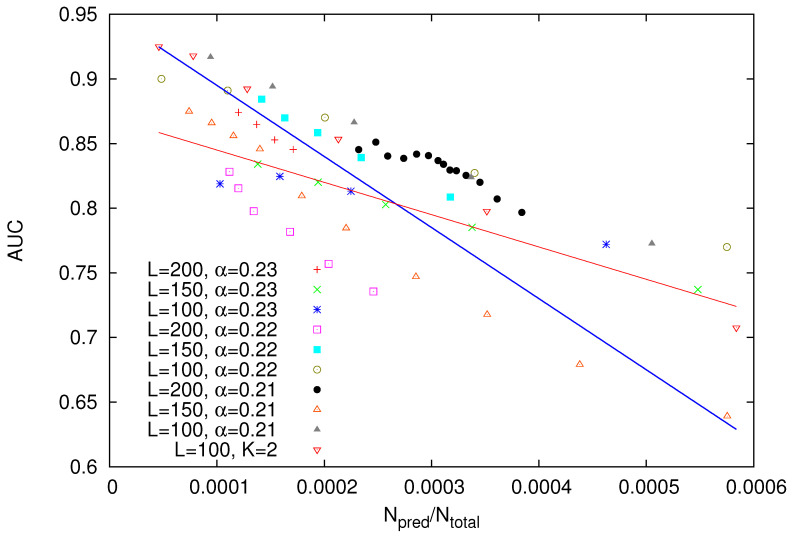
AUC obtained for the OFC EQ model with various values of the linear dimension L of the square lattice and parameters α or *K* versus the probability $\left(N_{pred}/N_{total}\right)$ of a strong avalanche to be observed. The blue straight line AUC $=0.95-550\times (N_{pred}/N_{total})$ has been drawn as a guide to the eye, while the thin red line AUC $=0.87-250\times (N_{pred}/N_{total})$ depicts a different linear trend exhibited by the data-points of α = 0.23 and either L = 100 or L = 150 (cf. the data-points of α = 0.22 and L = 100 show a similar trend but lie 0.05 above).

In Figure 10, we depict these values of the AUC as a function of the probability $N_{pred}/N_{total}$, where $N_{pred}$ stands for the number of the strong avalanches to be predicted, while $N_{total}$ is the total number of avalanches in the OFC model simulation. Interestingly, we observe that the predictability quantified by AUC increases when the rarity of the strong avalanches increase, i.e., $N_{pred}/N_{total}$ decreases. A close inspection of Figure 10 reveals that such a behaviour is observed by means of two distinct linear trends, see the blue and red straight lines in this figure. The lower trend (red straight line) is followed by the data-points of α=0.23 with *L* equal to either 150 or 100 and α=0.22 with *L* = 100, i.e., for the smallest linear sizes studied and the higher values of α, while the rest of the data-points follow the higher trend (blue straight line). These results are indicative of the complex influence of dissipation and finite size phenomena in the predictability of the OFC model. The fact that for the same value of *L* (*L* = 100), α=0.23 leads to 0.05 smaller AUC than that for α=0.22 (see the caption of Figure 10) is compatible with the observation by Pepke and Carlson [81] that the predictability in the OFC model diminishes as the level of conservation is increased.

## 4. Discussion

As mentioned in Section 2.4, Pasari [36] used the Log-normal distribution to model the interoccurrence natural time when employing nowcasting EQs [37] in the Bay of Bengal Region. Here, by using analytical (based on the Log-normal distribution) and numerical methods (the maximization with respect to *l* and *L*), we suggested appropriate bounds for the waiting (natural) time during which an alarm should be turned ON and attempted to generalize the nowcasting method to a forecasting one. The bounds have been selected so that the AUC of the corresponding ROC is maximum.

Concerning the OFC EQ model results shown in Figure 10, we observe that the quality of the predictions increases when the rarity of the strong avalanches increases (cf. this poses a constraint since only rare events are apt to prediction). Such a result, however, is not unexpected and corroborates Reference [101], where it has been shown that the stronger avalanches, deviating from the underlying power-law in self-organized critical systems, are the more susceptible ones to prediction.

Another point that should be mentioned is that in most of the cases shown in Figure 10, AUC ranges from 0.75 to 0.93 resulting from acceptable (0.7–0.8) to excellent (0.8–0.9) or even outstanding (0.9–1.0) performance [99,102,103]. Thus, we have obtained a reliable prediction method based solely on the avalanche sizes of the OFC EQ model. Interestingly, this not only holds for the OFC EQ model but it also holds for the real seismicity example of Figure 6 where AUC = 0.829 which is rated excellent according to the present standards [99,102,103].

Moreover, the procedure presented here has several advantages: The tools used are widely known in the scientific literature and do not require highly sophisticated methods or overly high computing power, this represents a great advantage since it means to be able to apply the procedure effectively and quickly. Another issue to highlight is that the present method, being more efficient for large events and therefore for more destructive EQs, may be of practical usefulness since it is for these events that it is desirable to have greater reliability. Of course, the constraints on the EQ catalog length, completeness, etc. that are necessary for nowcasting are still applicable. Finally, since for both, the synthetic case and for the case of real seismicity, the only knowledge required is the avalanche sizes or the seismic magnitudes, it is not necessary to know in great detail the conditions of the seismic region, a fact which facilitates and makes the application of the procedure more accessible (it seems that the information contained in other variables of the seismic region is encapsulated in the avalanche sizes time series). We feel that the present results may help on forecasting real EQs as well as understanding the phenomena precursory to strong EQs.

## 5. Conclusions

In summary, the following main results were obtained:

- Using the advantages of natural time analysis and the properties of the (natural interoccurrence) waiting time distribution, a method that generalizes EQ nowcasting to an EQ forecasting method has been presented.
- This forecasting method has been applied to a toy model which corresponds to the case when the waiting time distribution is Log-normal and the quality of the predictions was evaluated by the AUC in the ROC diagram. AUC has been estimated by means of the Log-normal distribution parameters as shown in Figure 4.
- The results for the Log-normal model have been applied to an example of real seismicity (i.e., the M${}_{w}$6.8 EQ that occurred in Greece on 25 October 2018 at 22:55 UTC) for which nowcasting has been already published [47] and it was found that this EQ could have been predicted with an FPr close to 0.25, while the corresponding AUC is 0.829.
- The forecasting method has been applied to the avalanches of the Olami-Feder-Christensen model leading to the AUC results shown in Figure 10. In this application, only the knowledge of the avalanche sizes was needed, while the force field $F_{i,j}$ was unknown, as in the case of real seismicity.

Most interestingly, both real seismicity and OFC model AUC results point to excellent or even outstanding quality of predictions.
